# An Ethnopharmaceutical Study on the Hypolipidemic Formulae in Taiwan Issued by Traditional Chinese Medicine Pharmacies

**DOI:** 10.3389/fphar.2022.900693

**Published:** 2022-09-15

**Authors:** Min-Han Chi, Jung Chao, Chien-Yu Ko, Shyh-Shyun Huang

**Affiliations:** ^1^ School of Pharmacy, China Medical University, Taichung, Taiwan; ^2^ Master Program for Food and Drug Safety, Chinese Medicine Research Center, Department of Chinese Pharmaceutical Sciences and Chinese Medicine Resources, China Medical University, Taichung, Taiwan; ^3^ Department of Food Nutrition and Health Biotechnology, Asia University, Taichung, Taiwan

**Keywords:** hyperlipidemia, hypolipidemic, traditional Chinese medicine, pharmacies, drug combination

## Abstract

Globally, approximately one-third of ischemic heart diseases are due to hyperlipidemia, which has been shown to cause various metabolic disorders. This study was aimed to disassemble and analyze hypolipidemic formulae sold by traditional Chinese medicine (TCM) pharmacies. Using commonly used statistical parameters in ethnopharmacology, we identified the core drug combination of the hypolipidemic formulae, thereby exploring the strategy by which the Taiwanese people select hypolipidemic drugs. Most important of all, we preserved the inherited knowledge of TCM. We visited 116 TCM pharmacies in Taiwan and collected 91 TCM formulae. The formulae were mainly disassembled by macroscopical identification, and the medicinal materials with a relative frequency of citation (RFC) >0.2 were defined as commonly used medicinal materials. Subsequently, we sorted the information of medicinal materials recorded in the Pharmacopeia, searched for modern pharmacological research on commonly used medicinal materials using PubMed database, and visualized data based on the statistical results. Finally, the core hypolipidemic medicinal materials used in folk medicine were obtained. Of the 91 TCM formulae collected in this study, 80 traditional Chinese medicinal materials were used, belonging to 43 families, predominantly Lamiaceae. Roots were the most commonly used part as a medicinal material. There were 17 commonly used medicinal materials. Based on medicinal records in Pharmacopeia, most flavors and properties were warm and pungent, the majority traditional effects were “tonifying and replenishing” and “blood-regulating.” Besides, the targeted diseases searching from modern pharmacological studies were diabetes mellitus and dyslipidemia. The core medicinal materials consisted of *Astragalus mongholicus* Bunge and *Crataegus pinnatifida* Bunge, and the core formulae were Bu-Yang-Huan-Wu-Tang and Xie-Fu-Zhu-Yu-Tang. In addition, 7 groups of folk misused medicinal materials were found. Although these TCMs have been used for a long period of time, their hypolipidemic mechanisms remain unclear, and further studies are needed to validate their safety and efficacy.

## 1 Introduction

Noncommunicable diseases (NCD), including heart diseases, strokes, cancer, and diabetes mellitus caused by hyperglycemia, and hyperlipidemia, carry common risk factors, such as tobacco smoking, alcoholism, and a sedentary lifestyle (World Health Organization, 2021a; World Health Organization, 2021b). Ischemic heart disease has been the leading cause of death globally for an extended period (World Health Organization, 2020; World Health Organization, 2021c). According to the World Health Organization (WHO) statistics, one-third of ischemic heart disease cases globally can be attributed to hyperlipidemia (World Health Organization, 2011). Many studies have shown that hyperlipidemia is a progression factor for incidence of coronary artery disease (CAD), atherosclerosis, and stroke (Yu et al., 2000; Kopin and Lowenstein, 2017). Besides, according to previous study, hyperlipidemia has a high correlation with pancreatitis, diabetes mellitus, and chronic kidney disease (CKD) (Rašlová, 2016; Hager et al., 2017; Athyros et al., 2018; Yang and McNabb-Baltar, 2020). Therefore, the American College of Cardiology (ACC) and American Heart Association (AHA) formulated treatment guidelines for lipid control, including lifestyle changes and drug treatment (Grundy et al., 2019; Reiter-Brennan et al., 2020), to prevent the occurrence of related diseases.

According to the treatment guidelines for lipid control, lifestyle changes are initially implemented for hyperlipidemia treatment, and the risk of cardiovascular diseases is continuously monitored. If the patient is at high risk for cardiovascular diseases or has familial hypercholesterolemia, drugs are included to control dyslipidemia (Grundy et al., 2019). Currently, statins are the mainstay of treatment for hyperlipidemia (Karr, 2017), where its mechanism involves the inhibition of HMG-CoA reductase to decrease blood lipid synthesis (Sirtori, 2014). Most patients use statins and are well tolerated. However, these drugs can cause side effects such as skeletal muscle pain, diabetes mellitus, and occurrence of central nervous system symptoms–statin-associated symptoms (SAS), which are commonly intolerable to patients (Thompson et al., 2016). Therefore, some patients also use other hypolipidemic agents (such as nicotinic acid, fibrates, bile acid sequestrant resin) with statins to control blood lipids effectively and reduce side effects (Karr, 2017).

According to statistics published by the Health Promotion Administration, Ministry of Health and Welfare in Taiwan, the prevalence of hyperlipidemia in people aged 18 years old and above from 2016 to 2019 was 21.63%, suggesting that every 1 out of 4–5 people suffer from hyperlipidemia (Health Promotion Administration, 2020). Nowadays, public acceptance of traditional Chinese medicine (TCM) has gradually increased (Sham et al., 2014; Hu and Wang, 2019). Although most people still comply with the treatment guidelines for dyslipidemia and use western medicine for lipid control, few people cannot tolerate the side effects of western medicine so that they change to use TCM combined with dietary control to treat dyslipidemia (Sham et al., 2014).

According to the theory of Chinese medicine, when “phlegm” and “dampness,” which are pathological products due to improper diet, generate in the body, if they are not handled properly, they will turn the body constitution into “qi stasis and blood stagnation” state, leading to abnormal blood circulation. If the situation continues, it will cause the weakness of liver, spleen and kidney. What’s worse, under the circumstances, “phlegm” and “dampness” are more likely to be generated, and such vicious circle will lead to the onset of hyperlipidemia (Shi and Li, 2007; O'Brien, 2010). In order to effectively eliminate “phlegm” and “dampness”, Chinese medical physician often use dampness-draining diuretic medicinal (e.g., Nelumbinis folium and Alismatis rhizoma) and blood-activating and stasis-dispelling medicinal (e.g., Persicae semen and Carthami flos) to improve body constitution so as to treat hyperlipidemia.

In addition to selecting TCM for regulating blood lipid level according to the theory of Chinese medicine, numerous modern pharmacological studies have shown that many TCMs exhibit hypolipidemic activity, such as Salviae miltiorrhizae radix et rhizoma, Crataegi fructus, Carthami flos, and Astragali radix (Guo et al., 2014; Liu J. et al., 2018; Fan et al., 2018). In addition, recent drug development has involved with extracting critical components from natural products, and western medicine formulations, such as capsules and pills, are used to improve the convenience of taking medicine and patient compliance. Well-known examples include extracting the active ingredient from *Monascus purpureus* Went., which was used to prepare a Chinese medicine capsule as a lipid-lowering agent; its main active ingredient is monacolin K (lovastatin), which is a statin drug. Although studies have shown its hypolipidemic effects, it can still cause severe side effects such as rhabdomyolysis (Lee et al., 2013). Therefore, modern research has focused on exploring other compounds in red yeast rice extract, such as monascin and ankaflavin (Lee et al., 2013; Lin et al., 2017; Xiong et al., 2019). In addition, studies have shown that monascin and ankaflavin have better hypolipidemic effects and lower toxicity compared with monacolin K (Lin et al., 2017).

Taiwanese people mainly obtain Chinese herbal medicines from health facilities (prescription Chinese herbal medicines) and TCM pharmacies (non-prescription Chinese herbal medicines). A study calculated the prevalence of Chinese herbal medicines purchased by Taiwanese people within 1 year and found that 74.8% of the public purchased non-prescription Chinese herbal medicines (Hu et al., 2020). With regards to this, TCM pharmacies seem to be the main suppliers for patients in Taiwan. However, types and dose ratios of medicinal materials, which are sold by different TCM pharmacies, differ. Recent studies analyzing hypolipidemic TCMs in Taiwan only used the National Health Insurance Research Database (NHIRD) to compile medication profiles (Chu et al., 2015) and did not examine the hypolipidemic formulae used by TCM pharmacies. In addition, a survey data from the Department of Statistics, Ministry of Health and Welfare revealed that the mean age of Chinese medicine distributors was 60 years, suggesting that the number of pharmacies and inherited knowledge related to TCM is gradually decreasing. Therefore, recording and preserving this crucial knowledge is necessary. The objective of this study was to use a systematic analysis to disassemble and analyze hypolipidemic formulae sold by TCM pharmacies and identify the core drug combination of hypolipidemic formulae, thereby exploring strategies by which the Taiwanese people select hypolipidemic drugs. Most importantly, we preserved the inherited traditional folk Chinese medicine knowledge.

## 2 Materials and Methods

### 2.1 Ethics Review

The study period was conducted from August 2020 to August 2021, and was approved by the Central Regional Research Ethics Committee of China Medical University, Taichung, Taiwan (CRREC-109-125) (Supplementary Figure S1).

### 2.2 Study Procedure

This study was divided into four stages: preparation of fieldwork, fieldwork, identification and preservation of medicinal materials, and analysis and sorting of medicinal material information. The detailed steps are shown in the flowchart (Supplementary Figure S2 **)**.

#### 2.2.1 Preparation of Fieldwork

Taiwan is a spindle-shaped island located in the west Pacific Ocean; its latitude range is 21°54′N–25°17′N, longitude range is 120°04′E–122°00′E, and the area is 35,873.196 km^2^. In this study, the statistical results of drug administration published by the Ministry of Health and Welfare (Ministry of Health and Welfare, 2021) were used to calculate the distribution ratios of TCM pharmacies in counties and cities of Taiwan. Set an anticipated number of formulae collected from each country and city. Finally, Google Maps was used to search and label the TCM pharmacies for the purpose of visit.

#### 2.2.2 Fieldwork

Visits were made to the labeled TCM pharmacies, hypolipidemic TCM formulae were purchased, and purchase information was recorded. If the formula purchase failed, the reason for failure was documented, and other local TCM pharmacies were visited immediately until all the expected number of formulae were collected. After the completion of this procedure, the formulae were disassembled in the lab.

#### 2.2.3 Identification and Preservation of Medicinal Materials

After the formula disassembly was completed, macroscopical identification was used to confirm the medicinal materials. The origin of each medicinal material was confirmed by Dr. Shyh-Shyun Huang (Associate professor of the School of Pharmacy, China Medical University, Taichung, Taiwan) and Dr. Jung Chao (Assistant Professor of the Department of Chinese Pharmaceutical Sciences and Chinese Medicine Resources, China Medical University, Taichung, Taiwan). After the medicinal materials were identified, they were photographed, numbered, and stored in the Dr. Shyh-Shyun Huang’s laboratory in China Medical University, Taichung, Taiwan.

#### 2.2.4 Analysis and Sorting of Medicinal Material Information

After identification of medicinal materials, the records of all collected medicinal materials obtained from various pharmacopeias were tallied. The pharmacopeias included Taiwan Herbal Pharmacopoeia 4^th^ Edition (Taiwan Herbal Pharmacopeia 4th Edition Committee, 2021), Pharmacopoeia of the People’s Republic of China 2020 Edition (Chinese Pharmacopoeia Commission, 2020), and Chinese Materia Medica (National Administration of Traditional Chinese Medicine “Chinese Materia Medica” Editorial Board, 1999). The material information was then sorted in terms of kingdom, local name, Latin name, scientific name, family, part used, traditional use, and flavors and properties. However, the literature used as a reference for data search and sorting in this study were pharmacy papers and their codification were not based on plant taxonomy. In order to facilitate subsequent data collection, the content in World Flora Online (Royal Botanic Gardens and Garden, 2021) was used as a standard, and the scientific name and family of medicinal materials were corrected.

Following this, we corrected misused items in all collected medicinal materials, and the ratios of authentic and misused items were calculated. In addition, the relative frequency of citation (RFC) of all collected medicinal materials was calculated using the following equation (Ahmad et al., 2017):

$$\mathrm{RFC}\;=\;\frac{\mathrm{the}\;\mathrm{number}\;\mathrm{of}\;\mathrm{informants}\;\mathrm{mentioning}\;\mathrm{specific}\;\mathrm{application}\;\mathrm{of}\;\mathrm{aspecies}\;}{\mathrm{the}\;\mathrm{total}\;\mathrm{number}\;\mathrm{of}\;\mathrm{informants}}$$

After referring to ethnopharmacology literature, medicinal materials with an RFC >0.2 were deemed to be commonly used medicinal materials (Chao et al., 2020; Ko et al., 2021; Su et al., 2021). Following this, the PubMed database was used to search for modern pharmacological research on these commonly used medicinal materials by using [(the scientific names of medicinal materials) and (Metabolic Diseases)] as keywords. The search period was from 2017 to 2021. If no relevant pieces of literature were found, a search was performed without date constraints.

According to the statistical variable classification, the appearance of medicinal materials was a categorical variable. For examining the correlation between the medicinal materials, their usage in each TCM pharmacy was used as a binary variable, and a 2 × 2 contingency table was constructed based on the number of occurrences of any two medicinal materials. The tabulated data were used to calculate the Phi correlation coefficient–a statistical parameter for measuring the correlation between binary variables. In this study, R software (version 4.1.1) was used to calculate Phi correlation coefficient. The range of the Phi correlation coefficient was between -1 and +1, suggesting that the closer the value is to the poles, the higher the correlation (positive or negative correlation). Finally, GraphPad Prism software (version 9.1.1) and Adobe illustrator (version 23.0.5) were used to make heat map and cladogram which were based on Phi correlation coefficient and RFC. The grid color in the heat map was based on the Phi correlation coefficient of two medicinal materials. The redder the color, the higher the correlation between the two medicinal materials. On the contrary, the bluer the color, the lower the correlation between the two medicinal materials. Through data visualization, the core medicinal materials were found out, and the composition of the hypolipidemic formulae commonly used by the public was concluded.

## 3 Results

### 3.1 Results of Hypolipidemic Formulae Collection and Analysis of Medicinal Material Information

**FIGURE 1:**
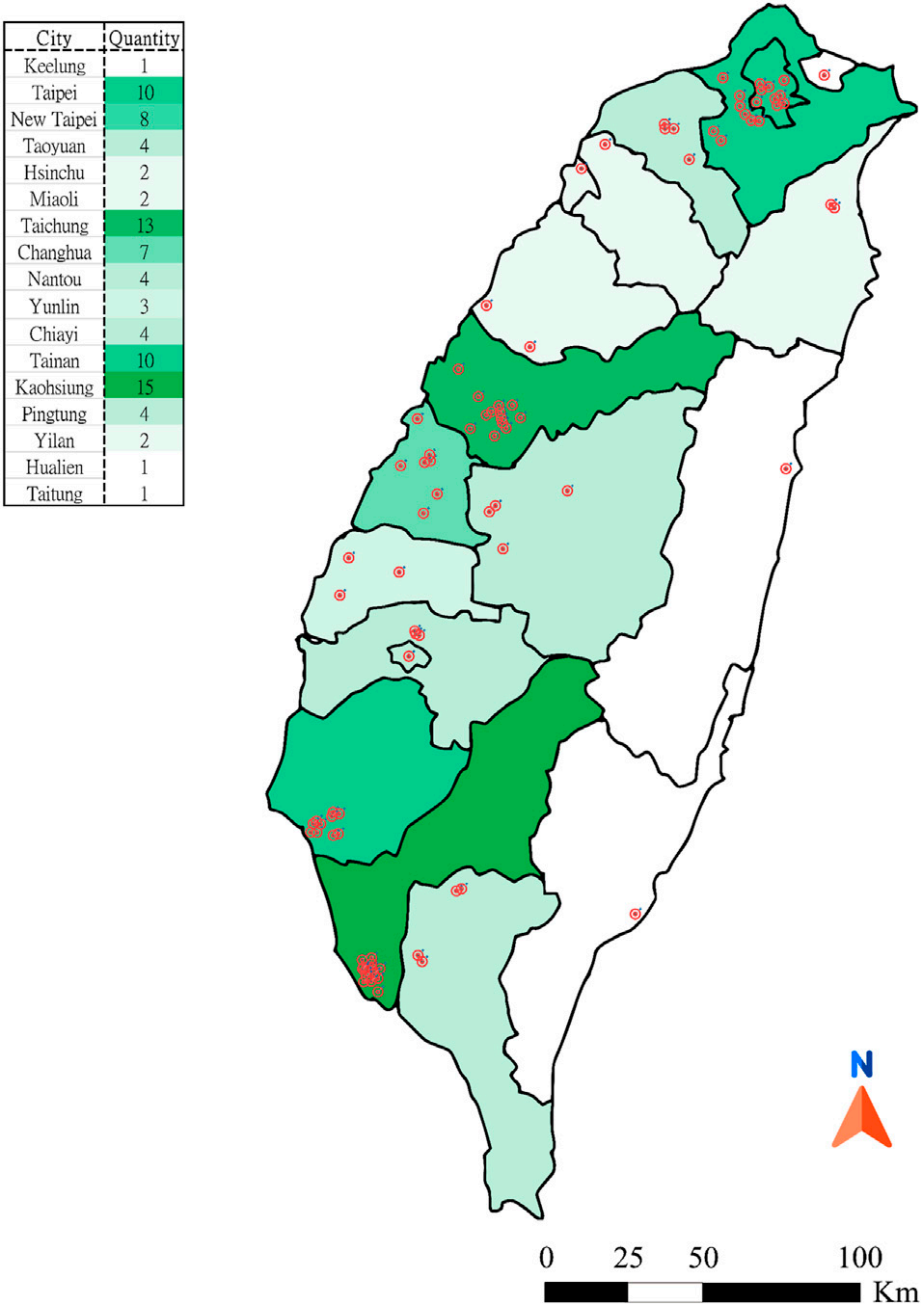
Distribution map of 91 hypolipidemic formulae.

According to data published by the Department of Statistics, Ministry of Health and Welfare in 2021, there were 9671 TCM pharmacies in Taiwan by the end of 2020, of which most were in Kaohsiung (n = 1,524, 15.65%) and the least in Taitung (n = 54, 0.56%) (Ministry of Health and Welfare, 2021). In this study, fieldwork was performed from August 2020 to August 2021, 116 TCM pharmacies were visited, of which most were in Kaohsiung (n = 15) and the least in Keelung (n = 1), Hualien (n = 1) and Taitung (n = 1). Finally, 91 hypolipidemic formulae were collected (Figure 1). In Figure 1, the distribution of red dots represents the location of pharmacies, and the greener the color of each city area, the more the number of pharmacies visited in the city.

**FIGURE 2:**
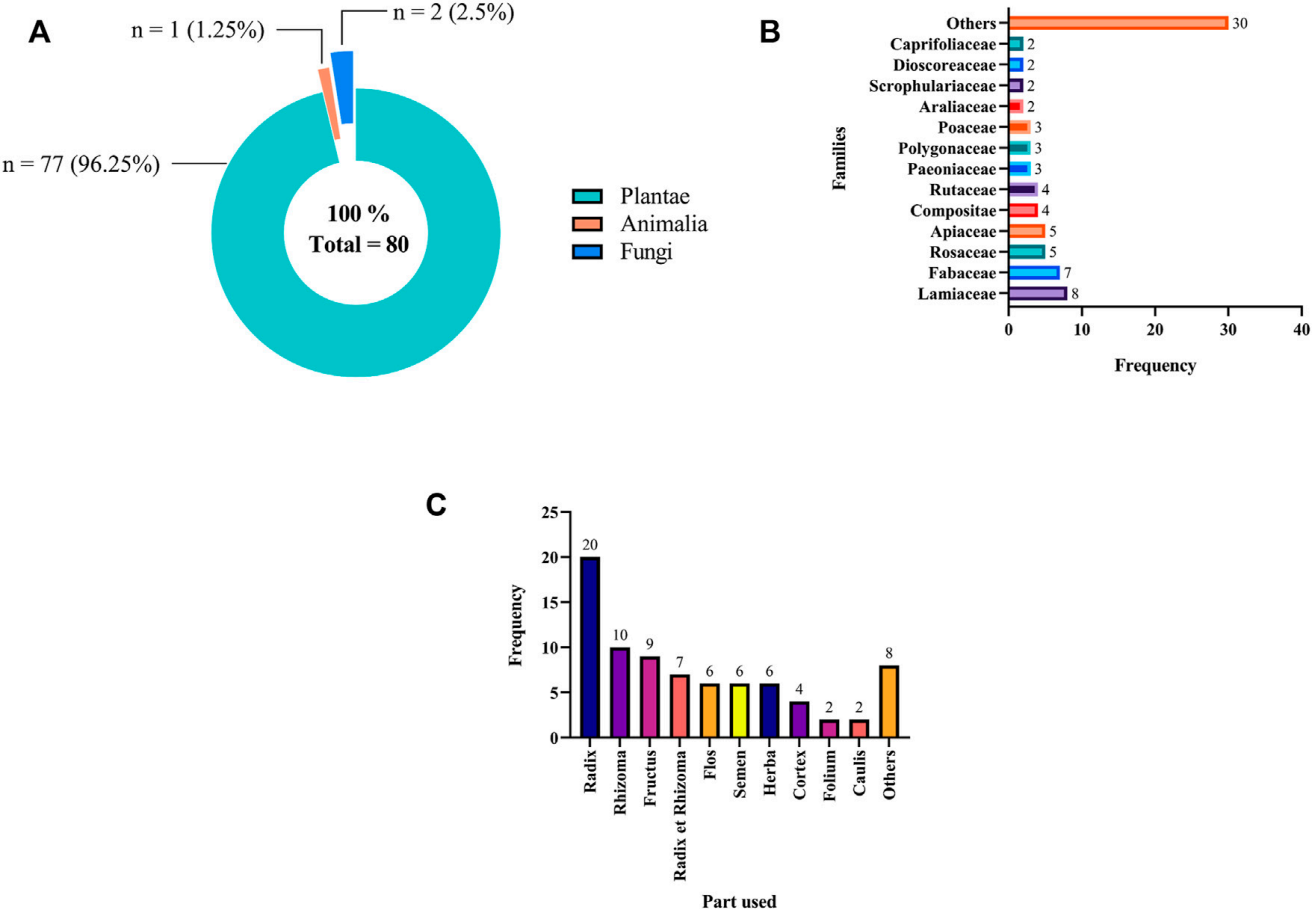
Classification of 80 TCM materials from 91 hypolipidemic formulae **(A)** kingdom **(B)** family **(C)** part used. Note: Parts with a frequency = 1 in the bar chart were combined in Others.

Statistical analysis of the disassembly results revealed that 80 TCM materials were collected in this study, of which 77 were plants (96.25%), two were fungi (2.5%), and one was an animal (1.25%) (Figure 2A). All collected medicinal materials were divided into 43 families, most of which were from Lamiaceae (frequency = 8), followed by Fabaceae (frequency = 7) (Figure 2B). Statistical results of the parts used suggested that roots (frequency = 20) were the most commonly used, followed by rhizomes (frequency = 10) **(** Figure 2C **)**.

### 3.2 Statistics of Core Medicinal Materials

**FIGURE 3:**
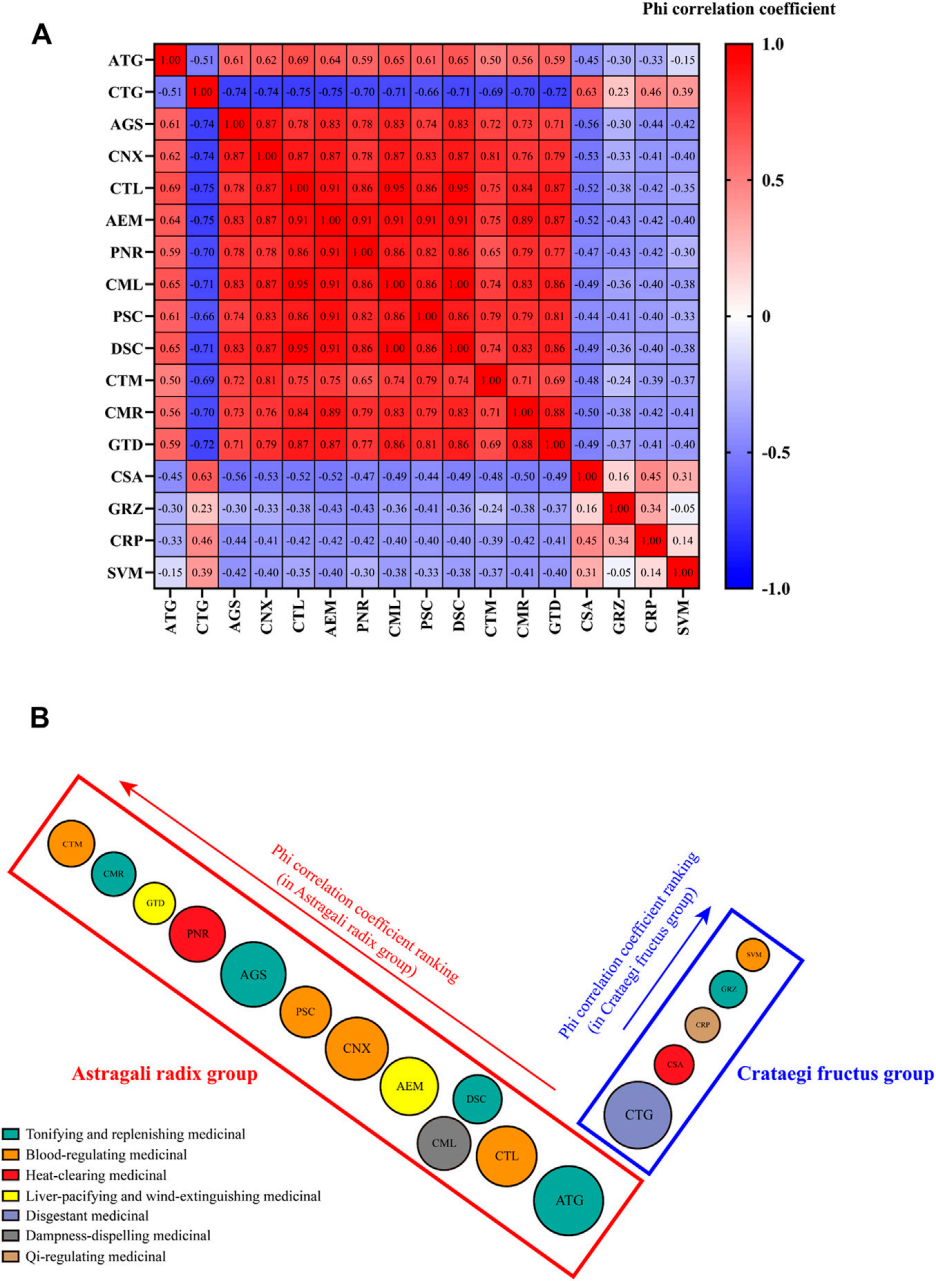
Correlation of 17 commonly used medicinal materials **(A)** Heat map **(B)** Cladogram. ATG, *Astragalus mongholicus* Bunge; AEM, *Amynthas aspergillum* (E. Perrier); AGS, *Angelica sinensis* (Oliv.) Diels; CML, *Chaenomeles speciosa* (Sweet) Nakai; CMR, *Cynomorium coccineum* subsp. *songaricum* (Rupr.) J.Léonard; CNX, *Ligusticum striatum* DC.; CRP, *Citrus reticulata* Blanco; CSA, *Senna obtusifolia* (L.) H. S. Irwin & Barneby; CTG, *Crataegus pinnatifida* Bunge; CTL, *Cyathula officinalis* K. C. Kuan; CTM, *Carthamus tinctorius* L.; DSC, *Dipsacus inermis* Wall.; GRZ, *Glycyrrhiza uralensis* Fisch.; GTD, *Gastrodia elata* Blume; PNR, *Paeonia lactiflora* Pall.; PSC, *Prunus persica* (L.) Batsch; SVM, *Salvia miltiorrhiza* Bunge.

The Phi correlation coefficients of the 17 commonly used medicinal materials were analyzed, of which one positive correlation group consisted of *Astragalus mongholicus* Bunge, *Angelica sinensis* (Oliv.) Diels, *Ligusticum striatum* DC., *Cyathula officinalis* K. C. Kuan, *Amynthas aspergillum* (E. Perrier), *Paeonia lactiflora* Pall., *Chaenomeles speciosa* (Sweet) Nakai, *Prunus persica* (L.) Batsch, *Dipsacus inermis* Wall., *Carthamus tinctorius* L., *Cynomorium coccineum* subsp*. songaricum* (Rupr.) J. Léonard, and *Gastrodia elata* Blume (known as the Astragali radix group). Another positive correlation group comprised *Crataegus pinnatifida* Bunge, *Senna obtusifolia* (L.) H. S. Irwin & Barneby, *Glycyrrhiza uralensis* Fisch., *Citrus reticulata* Blanco, and *Salvia miltiorrhiza* Bunge (known as Crataegi fructus group) (Figure 3A).

The Phi correlation coefficients of the Astragali radix group (compared with *Astragalus mongholicus* Bunge) listed in the descending order were *Astragalus mongholicus* Bunge (correlation coefficient = 1.000), *Cyathula officinalis* K. C. Kuan (correlation coefficient = 0.6851), *Chaenomeles speciosa* (Sweet) Nakai (correlation coefficient = 0.6540), *Dipsacus inermis* Wall. (correlation coefficient = 0.6540), *Amynthas aspergillum* (E. Perrier) (correlation coefficient = 0.6395), *Ligusticum striatum* DC. (correlation coefficient = 0.6152), *Prunus persica* (L.) Batsch (correlation coefficient = 0.6079), *Angelica sinensis* (Oliv.) Diels (correlation coefficient = 0.6051), *Paeonia lactiflora* Pall. (correlation coefficient = 0.5939), *Gastrodia elata* Blume (correlation coefficient = 0.5938), *Cynomorium coccineum* subsp*. songaricum* (Rupr.) J. Léonard (correlation coefficient = 0.5616), and *Carthamus tinctorius* L. (correlation coefficient = 0.4997). The Phi correlation coefficients of the Crataegi fructus group listed in the descending order (compared with *Crataegus pinnatifida* Bunge) were *Crataegus pinnatifida* Bunge (correlation coefficient = 1.000), *Senna obtusifolia* (L.) H. S. Irwin & Barneby (correlation coefficient = 0.6318), *Citrus reticulata* Blanco (correlation coefficient = 0.4615), *Salvia miltiorrhiza* Bunge (correlation coefficient = 0.3923), and *Glycyrrhiza uralensis* Fisch. (correlation coefficient = 0.2298). In addition, the Phi correlation coefficients of tonifying and replenishing medicinal and blood-regulating medicinal were the highest in the Astragali radix group; the Phi correlation coefficients of disgestant medicinal and heat-clearing medicinal were the highest in the Crataegi fructus group **(** Figure 3B **)**.

### 3.3 Sorting of Traditional Effects and Modern Pharmacological Research on Commonly Used Medicinal Materials

According to the literature, medicinal materials with RFC >0.2 are defined as commonly used materials (Chao et al., 2020; Ko et al., 2021; Su et al., 2021). In this study, a total of 17 of such materials have been investigated, which are ranked based on RFC in descending order: *Astragalus mongholicus* Bunge (RFC = 0.582), *Crataegus pinnatifida* Bunge (RFC = 0.516), *Angelica sinensis* (Oliv.) Diels (RFC = 0.462), *Ligusticum striatum* DC. (RFC = 0.440), *Cyathula officinalis* K. C. Kuan (RFC = 0.396), *Amynthas aspergillum* (E. Perrier) (RFC = 0.396), *Paeonia lactiflora* Pall. (RFC = 0.396), *Chaenomeles speciosa* (Sweet) Nakai (RFC = 0.374), *Prunus persica* (L.) Batsch (RFC = 0.374), *Dipsacus inermis* Wall. (RFC = 0.374), *Carthamus tinctorius* L. (RFC = 0.363), *Cynomorium coccineum* subsp*. songaricum* (Rupr.) J.Léonard (RFC = 0.341), *Gastrodia elata* Blume (RFC = 0.330), *Senna obtusifolia* (L.) H. S. Irwin & Barneby (RFC = 0.330), *Glycyrrhiza uralensis* Fisch. (RFC = 0.264), *Citrus reticulata* Blanco (RFC = 0.253), and *Salvia miltiorrhiza* Bunge (RFC = 0.242) (Table 1).

**TABLE 1:** Commonly used medicinal materials with RFC >0.2.

Latin name	Local name	Scientific name	Code / Voucher specimen	Family	Part used	RFC	Traditional use	Flavors / Properties	Literature on modern pharmacology research of dyslipidemia medicinal (PubMed Database)
Astragali radix	Huang ch’i 黃耆	*Astragalus mongholicus* Bunge	ATG / CRREC109125HLDATG53	Fabaceae	Radix	0.582	Tonifying and replenishing medicinal (Qi-tonifying medicinal)	Sweet / Warm	1. Renal injury (Zhang W. N. et al., 2018; Qin et al., 2020)
									2. Diabetes mellitus (Lien et al., 2016; Cao et al., 2017a; Behl and Kotwani, 2017; Chen et al., 2017; Nozaki et al., 2017; You et al., 2017; Zhang R. et al., 2018; Gao et al., 2018; He et al., 2018; Leng et al., 2018; Sun J. et al., 2019; Sun S. et al., 2019; Zhang R. et al., 2019; Jin et al., 2019; Liu et al., 2019; Zhai et al., 2019; Zheng et al., 2020a; Zheng et al., 2020b; Yang F. et al., 2020; Zhang R. et al., 2020; Zhang Y. et al., 2020; Sun H. H.et al., 2021; Zhou et al., 2021)
									3. Anti-cancer (You et al., 2017; Sun R. et al., 2021; Feng et al., 2021)
									4. Dyslipidemia (Fernandez et al., 2018; Sun J. et al., 2019; Zhang R. et al., 2020; Zhou et al., 2021)
									5. Obesity (Nie et al., 2018; Sun J. et al., 2019)
									6. Anti-oxidation (Chen et al., 2017; You et al., 2017; Leng et al., 2018; Jia N. et al., 2019; Zhang N. et al., 2019)
									7. Anti-inflammation (Cao et al., 2017a; Nikles et al., 2017; You et al., 2017; Fernandez et al., 2018; Leng et al., 2018; Jia N. et al., 2019; Zhang R. et al., 2019; Nöst et al., 2019; Zhang R. et al., 2020; Zhang Y. et al., 2020; Liu et al., 2021)
									8. Hypertension (You et al., 2017; Li et al., 2018)
									9. Osteoporosis (Sun N.Y. et al., 2021)
									10. Hepatic injury (Cao et al., 2017a; Chen Z. et al., 2019b; Zhou et al., 2021)
									11. Cardiovascular disease (Leng et al., 2018; Li et al., 2018)
Crataegi fructus	Shan cha 山楂	*Crataegus pinnatifida* Bunge	CTG / CRREC109125HLDCTG47	Rosaceae	Fructus	0.516	Disgestant medicinal	Sour, Sweet / Warm	1. Diabetes mellitus (Lee et al., 2016; Aierken et al., 2017; Dehghani et al., 2019; Hussain et al., 2021) 2. Dyslipidemia (Dehghani et al., 2019; Hussain et al., 2021) 3. Obesity (Lee et al., 2016; Dehghani et al., 2019; Hussain et al., 2021) 4. Hepatic injury (Hussain et al., 2021) 5. Cardiovascular disease (Dehghani et al., 2019)
Angelicae sinensis radix	Tang kuei 當歸	*Angelica sinensis* (Oliv.) Diels	AGS / CRREC109125HLDAGS42	Apiaceae	Radix	0.462	Tonifying and replenishing medicinal (Blood-tonifying medicinal)	Sweet, Pungent / Warm	1. Diabetes mellitus (Wang et al., 2016; Cao et al., 2017a; Huang F. et al., 2018; Soliman et al., 2019; Sui et al., 2019; Yang B. et al., 2020) 2. Anti-cancer (Yang B. et al., 2020; Feng et al., 2021) 3. Dyslipidemia (Wang et al., 2016; Wu et al., 2016) 4. Anti-oxidation (Yang B. et al., 2020) 5. Anti-inflammation (Cao et al., 2017a; Hua et al., 2019; Yang B. et al., 2020) 6. Osteoporosis (Liao F. et al., 2019; Xie et al., 2019; Yang et al., 2019) 7. Hepatic injury (Wang et al., 2016; Cao et al., 2017a) 8. Cardiovascular disease (Wu et al., 2016)
Chuanxiong rhizoma	Ch’uan ch’iung 川芎	*Ligusticum striatum* DC.	CNX / CRREC109125HLDCNX40	Apiaceae	Rhizoma	0.44	Blood-regulating f and stasis-dispelling medicinal)	Pungent / Warm	1. Diabetes mellitus (Yang et al., 2018; Rai et al., 2019) 2. Dyslipidemia (Dong et al., 2020) 3. Anti-oxidation (Ge et al., 2018; Zhou Q. et al., 2020; Dong et al., 2020) 4. Anti-inflammation (Rai et al., 2019) 5. Hypertension (Gao et al., 2019) 6. Osteoporosis (Yang D. et al., 2020; Dong et al., 2020) 7. Cardiovascular disease (Zhou Q. et al., 2020)
Cyathulae radix	Ch’uan niu hsi 川牛膝	*Cyathula officinalis* K.C.Kuan	CTL / CRREC109125HLDCTL36	Amaranthaceae	Radix	0.396	Blood-regulating medicinal (Blood-activating and stasis-dispelling medicinal)	Bitter, Sour / Plain	1. Anti-oxidation (Cao et al., 2017b) 2. Anti-inflammation (Cao et al., 2017b; Feng et al., 2017; Feng et al., 2019) 3. Hepatic injury (Meng et al., 2019) 4. Cardiovascular disease (Cao et al., 2017b; Zhao et al., 2017)
Amynthas et metaphire	Ti lung 地龍	*Amynthas aspergillum* (E.Perrier)	AEM / CRREC109125HLDAEM36	Megascolecidae	Dried body	0.396	Liver-pacifying and wind-extinguishing medicinal	Salty / Cold	None
Paeoniae radix rubra	Ch’ih shao 赤芍	*Paeonia lactiflora* Pall.	PNR / CRREC109125HLDPNR36	Paeoniaceae	Radix	0.396	Heat-clearing medicinal (Heat-clearing and blood-cooling medicinal)	Bitter / Cold	1. Diabetes mellitus (Zhu et al., 2016; Sun et al., 2017; Zhong et al., 2017; Liao W. T. et al., 2019) 2. Dyslipidemia (Hu et al., 2017) 3. Obesity (Zhong et al., 2017) 4. Anti-oxidation (Hu et al., 2017; Xia et al., 2017) 5. Anti-inflammation (Zhu et al., 2016; Xia et al., 2017; Nöst et al., 2019) 6. Hepatic injury (Xia et al., 2017)
Chaenomelis fructus	Mu kua 木瓜	*Chaenomeles speciosa* (Sweet) Nakai	CML / CRREC109125HLDCML34	Rosaceae	Fructus	0.374	Dampness-dispelling medicinal (Wind-dampness-dispelling medicinal)	Sour / Warm	1. Diabetes mellitus (Zheng X. et al., 2018; Huang W. et al., 2018; Deng et al., 2020; Turkiewicz et al., 2020) 2. Anti-cancer (Huang W. et al., 2018; Cheng et al., 2020b) 3. Dyslipidemia (Huang W. et al., 2018) 4. Anti-oxidation (Xie et al., 2016; Miao et al., 2017; Zheng X. W. et al., 2018; Huang W. et al., 2018; Ma J. et al., 2019; Hendrich et al., 2020; Turkiewicz et al., 2020) 5. Anti-inflammation (Ma J. et al., 2019; Wang Z. J. et al., 2021) 6. Hypertension (Huang W. et al., 2018)
Persicae semen	T’ao jên 桃仁	*Prunus persica* (L.) Batsch	PSC / CRREC109125HLDPSC34	Rosaceae	Semen	0.374	Blood-regulating medicinal (Blood-activating and stasis-dispelling medicinal)	Bitter, Sweet / Plain	1. Diabetes mellitus (Jung et al., 2017; Wang et al., 2017; Nowicka et al., 2018) 2. Dyslipidemia (Jung et al., 2017) 3. Obesity (Nowicka et al., 2018) 4. Anti-oxidation (Nowicka et al., 2018) 5. Cardiovascular disease (Ren et al., 2017)
Dipsaci radix	Hsü tuan 續斷	*Dipsacus inermis* Wall.	DSC / CRREC109125HLDDSC34	Caprifoliaceae	Radix	0.374	Tonifying and replenishing medicinal (Yang-tonifying medicinal)	Bitter, Pungent / Warm	1. Anti-inflammation (Hassan et al., 2020) 2. Osteoporosis (He et al., 2019)
Carthami flos	Hung hua 紅花	*Carthamus tinctorius* L.	CTM / CRREC109125HLDCTM33	Compositae	Flos	0.363	Blood-regulating medicinal (Blood-activating and stasis-dispelling medicinal)	Pungent / Warm	1. Renal injury (Qin et al., 2020) 2. Diabetes mellitus (Li et al., 2017; Liu J. et al., 2018; Xu et al., 2018; Lee M. et al., 2020a; Lee M. et al., 2020b; Li S. R. et al., 2020; Orgah et al., 2020) 3. Anti-cancer (Orgah et al., 2020) 4. Dyslipidemia (Fan et al., 2018; Lee M. et al., 2020a; Lee M. et al., 2020b; Nimrouzi et al., 2020) 5. Obesity (Liu J. et al., 2018) 6. Anti-oxidation (Wu et al., 2018; Xu et al., 2018; Lee M. et al., 2020b; Nimrouzi et al., 2020) 7. Anti-inflammation (Han et al., 2016; Liu J. et al., 2018; Lee M. et al., 2020b; Nimrouzi et al., 2020; Orgah et al., 2020) 8. Osteoporosis (Choi et al., 2017; Liu L. et al., 2018) 9. Cardiovascular disease (Han et al., 2016; Fan et al., 2018; Meng et al., 2018; Wu et al., 2018; Meng et al., 2020; Orgah et al., 2020)
Cynomorii herba	So yang 鎖陽	*Cynomorium coccineum subsp. songaricum* (Rupr.) J.Léonard	CMR / CRREC109125HLDCMR31	Cynomoriaceae	Herba	0.341	Tonifying and replenishing medicinal (Yang-tonifying medicinal)	Sweet / Warm	1. Diabetes mellitus (Shi et al., 2021)
Gastrodiae rhizoma	T’ien ma 天麻	*Gastrodia elata* Blume	GTD / CRREC109125HLDGTD30	Orchidaceae	Rhizoma	0.33	Liver-pacifying and wind-extinguishing medicinal	Sweet / Plain	1. Diabetes mellitus (Ye et al., 2018) 2. Dyslipidemia (Ye et al., 2018) 3. Anti-inflammation (Ye et al., 2018) 4. Hypertension (Gao et al., 2019) 5. Osteoporosis (Liu S. et al., 2018)
Cassiae semen	Chüeh ming tzu 決明子	*Senna obtusifolia* (L.) H.S.Irwin & Barneby	CSA / CRREC109125HLDCSA30	Fabaceae	Semen	0.33	Heat-clearing medicinal (Heat-clearing and fire-purging medicinal)	Sweet, Bitter, Salty / Cold	1. Diabetes mellitus (Jung et al., 2016; Subash-Babu and Alshatwi, 2018; Wang et al., 2019; Ko et al., 2020) 2. Dyslipidemia (Kambalachenu et al., 2018) 3. Obesity (Wang et al., 2019) 4. Anti-oxidation (Kambalachenu et al., 2018; Tang et al., 2018) 5. Anti-inflammation (Subash-Babu and Alshatwi, 2018; Wang et al., 2019)
Glycyrrhizae radix et rhizoma	Kan ts’ao 甘草	*Glycyrrhiza uralensis* Fisch.	GRZ / CRREC109125HLDGRZ24	Fabaceae	Radix et Rhizoma	0.264	Tonifying and replenishing medicinal (Qi-tonifying medicinal)	Sweet / Plain	1. Renal injury (Cheng et al., 2020a) 2. Diabetes mellitus (Zhang Y. et al., 2016; Rani et al., 2017; Ryuk et al., 2017; Huang F. et al., 2018; Lee H. E. et al., 2018; Wang et al., 2018; Alam et al., 2019; Bai et al., 2019; Carnovali et al., 2019; Luo et al., 2019; Yamashita et al., 2019; Alzahrani et al., 2020; Yang L. et al., 2020; Yang M. et al., 2020; Fan et al., 2020; Xu et al., 2020; Igarashi et al., 2021) 3. Anti-cancer (Zhang Y. et al., 2016) 4. Dyslipidemia (Lee E. J. et al., 2018; Lee H. E. et al., 2018; Cheng et al., 2020a; Yang M. et al., 2020; Igarashi et al., 2021) 5. Obesity (Lee H. E.et al., 2018; Igarashi et al., 2021) 6. Anti-oxidation (Zhang Y. et al., 2016; Sil and Chakraborti, 2016; Carnovali et al., 2019; Cheng et al., 2020a; Alzahrani et al., 2020; Baek et al., 2020; Li X. et al., 2020; Yang L. et al., 2020) 7. Anti-inflammation (Zhang Y. et al., 2016; Carnovali et al., 2019; Nöst et al., 2019; Cheng et al., 2020a; Alzahrani et al., 2020; Baek et al., 2020) 8. Osteoporosis (Rho et al., 2017; Carnovali et al., 2019; Carnovali et al., 2020) 9. Hepatic injury (Zhang E. et al., 2016; Sil and Chakraborti, 2016; Lee E. J. et al., 2018; Baek et al., 2020)
Citri reticulatae pericarpium	Chü p’i 橘皮	*Citrus reticulata* Blanco	CRP / CRREC109125HLDCRP23	Rutaceae	Pericarpium	0.253	Qi-regulating medicinal	Bitter, Pungent / Warm	1. Diabetes mellitus (Lu H. L. et al., 2020; Dhuique-Mayer et al., 2020; Gandhi et al., 2020; Guo et al., 2020; Kong et al., 2020; Mato Mofo et al., 2020; Yousof Ali et al., 2020; Al-Aubaidy et al., 2021; Li et al., 2021; Meephat et al., 2021; Naeini et al., 2021) 2. Anti-cancer (Song T. et al., 2020) 3. Dyslipidemia (Castro et al., 2020; Dhuique-Mayer et al., 2020; Kong et al., 2020; Li et al., 2021; Meephat et al., 2021; Naeini et al., 2021) 4. Obesity (Lu J. F. et al., 2020; Mato Mofo et al., 2020; Dincer and Yuksel, 2021; Naeini et al., 2021; Testai et al., 2021) 5. Anti-oxidation (Castro et al., 2020; Kong et al., 2020; Yousof Ali et al., 2020; Al-Aubaidy et al., 2021; Wang M. et al., 2021; Naeini et al., 2021) 6. Anti-inflammation (Lu J. F. et al., 2020; Al-Aubaidy et al., 2021; Meephat et al., 2021; Naeini et al., 2021; Testai et al., 2021) 7. Hypertension (Dhuique-Mayer et al., 2020; Meephat et al., 2021) 8. Hepatic injury (Naeini et al., 2021) 9. Cardiovascular disease (Castro et al., 2020; Li et al., 2021; Meephat et al., 2021; Testai et al., 2021)
Salviae miltiorrhizae radix et rhizoma	Tan shên 丹參	*Salvia miltiorrhiza* Bunge	SVM / CRREC109125HLDSVM22	Lamiaceae	Radix et Rhizoma	0.242	Blood-regulating medicinal (Blood-activating and stasis-dispelling medicinal)	Bitter / Cold	1. Renal injury (Qin et al., 2020) 2. Diabetes mellitus (Behl and Kotwani, 2017; Chen L. et al., 2019; Jia Q. et al., 2019; Ma L. et al., 2019; Cheng et al., 2019; Zhao et al., 2019; Li C. L. et al., 2020; Lu H. L. et al., 2020; Song M. et al., 2020; Zhang B. et al., 2020; Zheng et al., 2020a; Zheng et al., 2020b; Zhou J. et al., 2020; Orgah et al., 2020; Singh et al., 2020; Wang et al., 2020; Abd Rashed and Rathi, 2021; Sun H. H. et al., 2021; Guo et al., 2021; Huang et al., 2021; Yin et al., 2021) 3. Anti-cancer (Fürstenau et al., 2019; Shi et al., 2019; Lu J. F.et al., 2020; Orgah et al., 2020) 4. Dyslipidemia (Fan et al., 2018; Ma L. et al., 2019; Ma et al., 2020; Huang et al., 2021; Yin et al., 2021; Zhang et al., 2021) 5. Obesity (An et al., 2019; Ma L. et al., 2019; Cheng et al., 2019; Huang et al., 2021) 6. Anti-oxidation (Chen L. et al., 2019; Fürstenau et al., 2019; Shi et al., 2019; Zhao et al., 2019; Zhang B. et al., 2020; Zhou J. et al., 2020; Du et al., 2020; Yin et al., 2021) 7. Anti-inflammation (Shi et al., 2019; Du et al., 2020; Ma et al., 2020; Orgah et al., 2020; Wang et al., 2020; Huang et al., 2021; Yin et al., 2021) 8. Hypertension (Zhou J. et al., 2020) 9. Osteoporosis (He et al., 2019; Zhang J. et al., 2020; Lee S. R. et al., 2020) 10. Hepatic injury (Wang et al., 2020) 11. Cardiovascular disease (Fan et al., 2018; Ma L. et al., 2019; Zhou J. et al., 2020; Du et al., 2020; Hao et al., 2020; Kumar et al., 2020; Ma et al., 2020; Orgah et al., 2020; Sun et al., 2020; Guan and Wang, 2021; Yin et al., 2021; Zhang et al., 2021)

**FIGURE 4:**
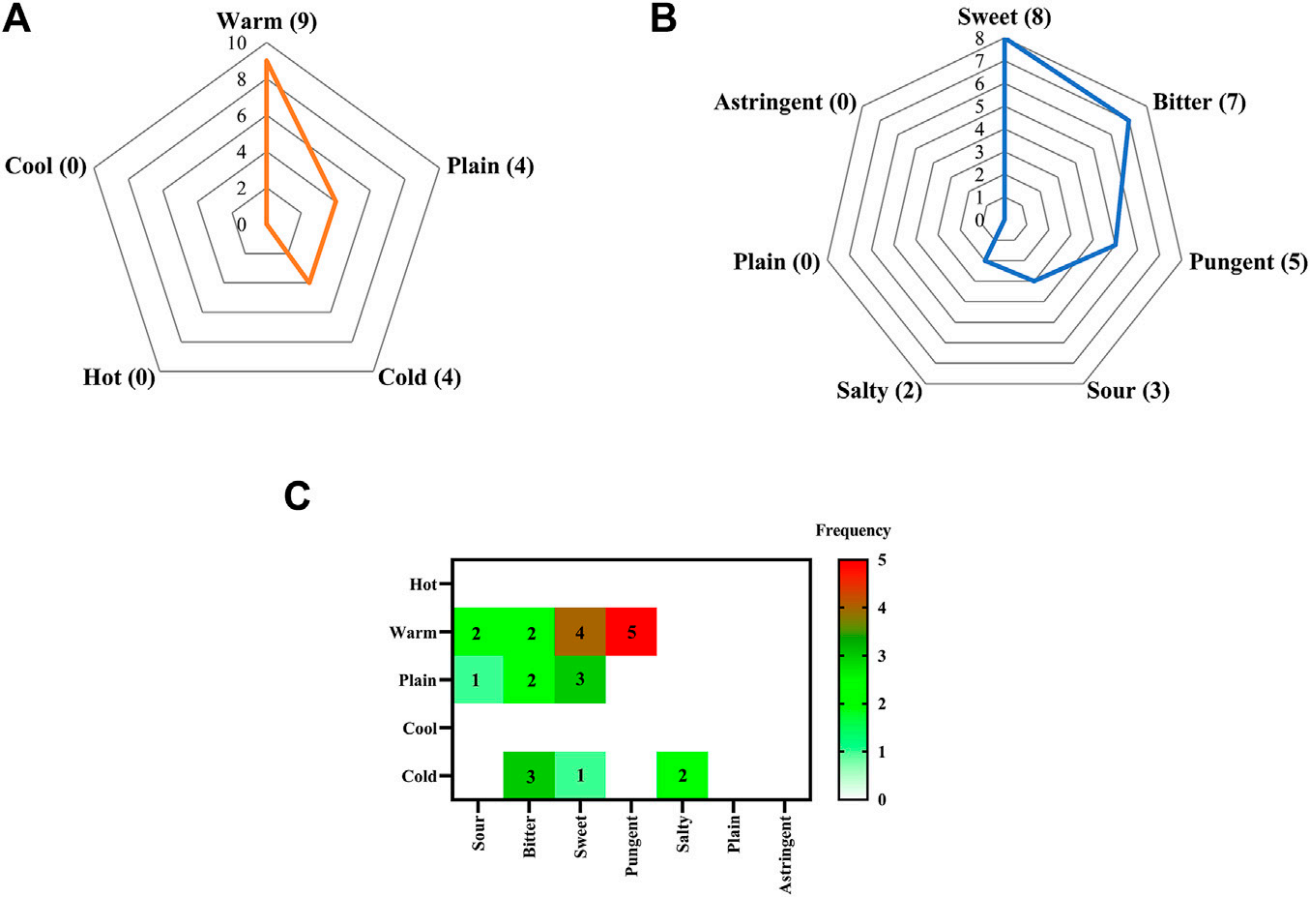
Flavor and property statistics and analysis charts of 17 commonly used medicinal materials **(A)** Property radar plot **(B)** Flavor radar plot **(C)** Correlation heat map of flavors and properties.

With regards to property and flavor, the most common property of the 17 commonly used medicinal materials was warm (frequency = 9), followed by plain (frequency = 4), and the most common flavor was sweet (frequency = 8), followed by bitter (frequency = 7) (Figures 4A,B). Combining the two factors, i.e., properties and flavors, most drugs were warm and pungent (frequency = 5), followed by sweet and warm (frequency = 4) (Figure 4C).

**FIGURE 5:**
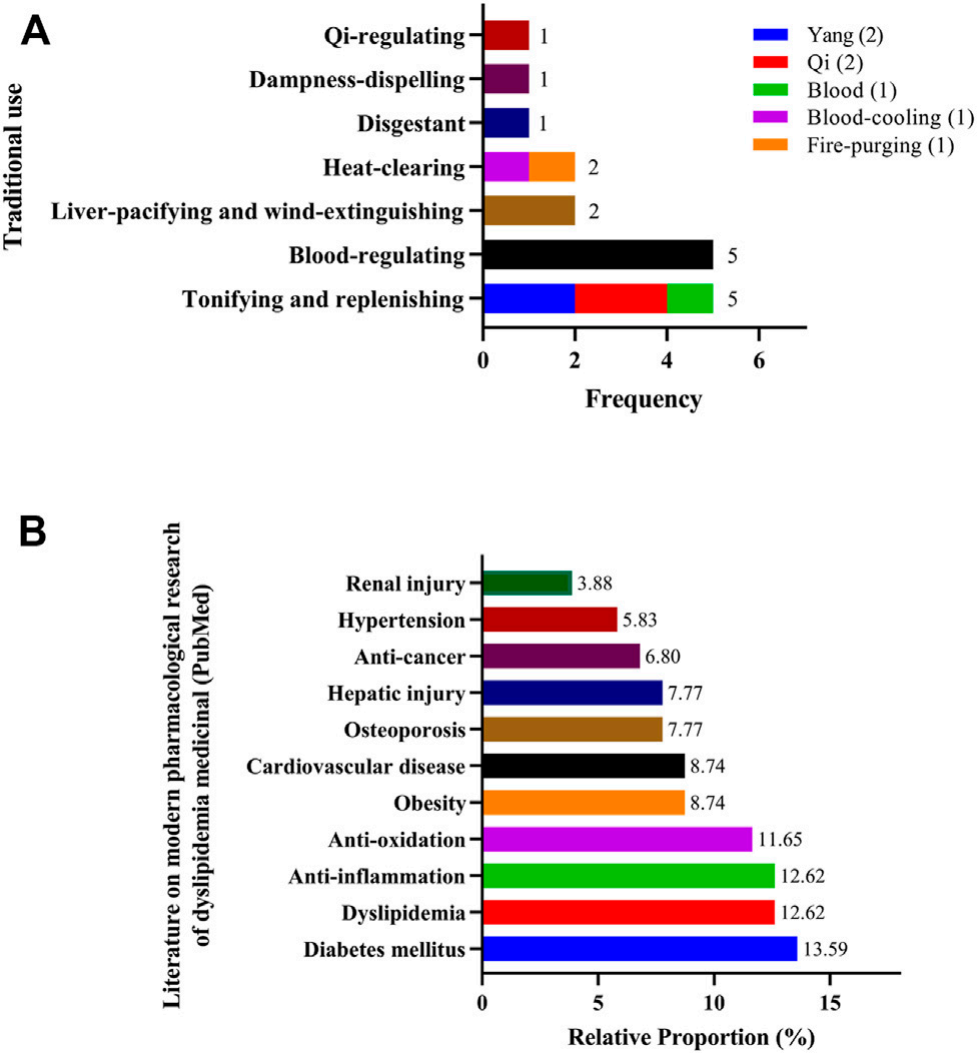
Purpose statistics of 17 commonly used medicinal materials **(A)** Traditional use statistics **(B)** Modern pharmacological research statistics.

The 17 commonly used medicinal materials can be divided into seven categories based on their traditional use, namely tonifying and replenishing medicinal (frequency = 5), blood-regulating medicinal (frequency = 5), liver-pacifying and wind-extinguishing medicinal (frequency = 2), heat-clearing medicinal (frequency = 2), disgestant medicinal (frequency = 1), dampness-dispelling medicinal (frequency = 1), and qi-regulating medicinal (frequency = 1). Among these categories, tonifying and replenishing medicinal can be subdivided into yang-tonifying medicinal (frequency = 2), qi-tonifying (frequency = 2), and blood-tonifying medicinal (frequency = 1); heat-clearing medicinal can be subdivided into heat-clearing and blood-cooling medicinal (frequency = 1) and heat-clearing and fire-purging medicinal (frequency = 1) (Figure 5A).

With regard to modern pharmacological research, search results from PubMed database showed that the therapeutic purpose of 17 commonly used medicinal materials was distributed in 11 diseases or physiological responses, which in descending order were diabetes mellitus (13.59%), dyslipidemia (12.62%), anti-inflammation (12.62%), anti-oxidation (11.65%), obesity (8.74%), cardiovascular disease (8.74%), osteoporosis (7.77%), hepatic injury (7.77%), anti-cancer (6.80%), hypertension (5.83%), and renal injury (3.88%) (Figure 5B).

### 3.4 Statistics of Authentic and Misused Medicinal Materials

**FIGURE 6:**
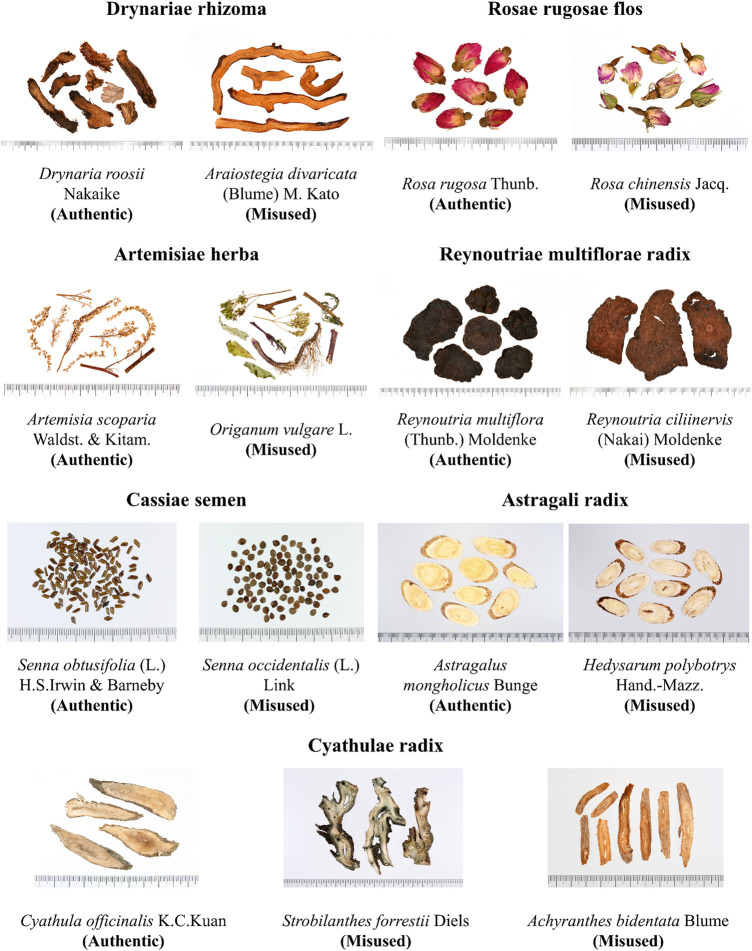
Photograph of authentic and misused medicinal materials.

Of the 91 hypolipidemic formulae collected in this study, there were seven groups of authentic and misused medicinal materials (Table 2; Figure 6):

1. *Drynaria roosii* Nakaike (Authentic, 0%) and *Araiostegia divaricata* (Blume) M. Kato (Misused, 100%)
2. *Rosa rugosa* Thunb. (Authentic, 20%) and *Rosa chinensis* Jacq. (Misused, 80%)
3. *Artemisia scoparia* Waldst. & Kitam. (Authentic, 0%), *Origanum vulgare* L. (Misused, 100%)
4. *Cyathula officinalis* K. C. Kuan (Authentic, 6.82%), *Strobilanthes forrestii* Diels (Misused, 75%), and *Achyranthes bidentata* Blume (Misused, 18.18%)
5. *Astragalus mongholicus* Bunge (Authentic, 1.85%) and *Hedysarum polybotrys* Hand.-Mazz. (Misused, 98.15%)
6. *Senna obtusifolia* (L.) H. S. Irwin & Barneby (Authentic, 93.33%), *Senna occidentalis* (L.) Link (Misused, 6.67%)
7. *Reynoutria multiflora* (Thunb.) Moldenke (Authentic, 23.08%) and *Reynoutria ciliinervis* (Nakai) Moldenke (Misused, 76.92%)

**TABLE 2:** Analysis of authentic and misused medicinal materials

Latin name	Authentic					Misused				
	Local name	Scientific name	Family	Part used	Frequency (ratio)	Local name	Scientific name	Family	Part used	Frequency (ratio)
Drynariae rhizoma	Ku sui pu 骨碎補	*Drynaria roosii* Nakaike	Polypodiaceae	Rhizoma	0 (0%)	Ta yeh ku sui pu 大葉骨碎補	*Araiostegia divaricata* (Blume) M. Kato	Davalliaceae	Rhizoma	1 (100%)
Rosae rugosae flos	Mei kuei 玫瑰	*Rosa rugosa* Thunb.	Rosaceae	Flos	1 (20%)	Yüeh chi 月季	*Rosa chinensis* Jacq.	Rosaceae	Flos	4 (80%)
Artemisiae herba	Yin ch'ên 茵陳	*Artemisia scoparia* Waldst. & Kitam.	Compositae	Herba	0 (0%)	Niu chih 牛至	*Origanum vulgare* L.	Lamiaceae	Herba	2 (100%)
Cyathulae radix	Ch’uan niu hsi 川牛膝	*Cyathula officinalis* K.C.Kuan	Amaranthaceae	Radix	3 (6.82%)	Wei niu hsi 味牛膝	*Strobilanthes forrestii* Diels	Acanthaceae	Radix	33 (75%)
						Niu hsi 牛膝	*Achyranthes bidentata* Blume	Amaranthaceae	Radix	8 (18.18%)
Astragali radix	Huang ch’i 黃耆	*Astragalus mongholicus* Bunge	Fabaceae	Radix	1 (1.85%)	Hung ch’i 紅耆	*Hedysarum polybotrys* Hand.-Mazz.	Fabaceae	Radix	53 (98.15%)
Cassiae semen	Chüeh ming tzu 決明子	*Senna obtusifolia* (L.) H.S.Irwin & Barneby	Fabaceae	Semen	28 (93.33%)	Wang chiang nan 望江南	*Senna occidentalis* (L.) Link	Fabaceae	Semen	2 (6.67%)
Reynoutriae multiflorae radix	Hê shou wu 何首烏	*Reynoutria multiflora* (Thunb.) Moldenke	Polygonaceae	Radix	3 (23.08%)	I liao 翼蓼	*Reynoutria ciliinervis* (Nakai) Moldenke	Polygonaceae	Radix	10 (76.92%)

## 4 Discussion

### 4.1 Analysis of the Corresponding Prescriptions of Core Medicinal Materials

In this study, the core hypolipidemic formulae could be classified into two medicinal material combinations: The first comprising 12 medicinal materials in which *Astragalus mongholicus* Bunge was the dominant and contained *Astragalus mongholicus* Bunge, *Cyathula officinalis* K. C. Kuan, *Chaenomeles speciosa* (Sweet) Nakai, *Dipsacus inermis* Wall., *Amynthas aspergillum* (E. Perrier), *Ligusticum striatum* DC., *Prunus persica* (L.) Batsch, *Angelica sinensis* (Oliv.) Diels, *Paeonia lactiflora* Pall., *Gastrodia elata* Blume, *Cynomorium coccineum* subsp*. songaricum* (Rupr.) J. Léonard, and *Carthamus tinctorius* L. These formulae included Bu-Yang-Huan-Wu-Tang [*Astragalus mongholicus* Bunge, *Angelica sinensis* (Oliv.) Diels, *Paeonia lactiflora* Pall., *Amynthas aspergillum* (E. Perrier), *Ligusticum striatum* DC., *Prunus persica* (L.) Batsch, *Carthamus tinctorius* L.] (Zheng XW. et al., 2018), and Xie-Fu-Zhu-Yu-Tang [*Angelica sinensis* (Oliv.) Diels, *Rehmannia glutinosa* (Gaertn.) DC., *Prunus persica* (L.) Batsch, *Carthamus tinctorius* L., *Citrus × aurantium* L., *Paeonia lactiflora* Pall., *Bupleurum chinense* DC., *Glycyrrhiza uralensis* Fisch., *Platycodon grandiflorus* (Jacq.) A. DC., *Ligusticum striatum* DC., and *Achyranthes bidentata* Blume] (Wang and Qiu, 2019). The second combination contained five medicinal materials in which *Crataegus pinnatifida* Bunge was the dominant, and comprised of *Crataegus pinnatifida* Bunge, *Senna obtusifolia* (L.) H. S. Irwin & Barneby, *Citrus reticulata* Blanco, *Salvia miltiorrhiza* Bunge, and *Glycyrrhiza uralensis* Fisch. Based on previous studies, this combination of medicinal materials is often used in hypolipidemic control (Yin and He, 2015).

### 4.2 Correlation Analysis of Core Medicinal Materials

To further confirm the traditional use distribution and composition of hypolipidemic formulae, two medicinal materials, *Astragalus mongholicus* Bunge and *Crataegus pinnatifida* Bunge with high RFC and negative correlation with each other, were used to construct cladograms based on Phi correlation coefficients. The size of the circles represents the magnitude of the RFC value, and different colors represent different traditional uses. From the cladograms, it can be seen that medicinal materials with higher RFC may not necessarily have higher correlation coefficients. In addition, Phi correlation coefficient analysis show that *Chaenomeles speciosa* (Sweet) Nakai and *Dipsacus inermis* Wall have the same correlation coefficient, showing that these two drugs are a pair that will simultaneously occur in hypolipidemic formulae.

### 4.3 Modern Research on the Flavor and Property of Core Medicinal Materials

Each medicinal material might incorporate many flavors and only one property. The properties can be classified as cold, cool, plain, warm, and hot, of which cool and cold are in one group, and warm and hot are in another. The two concepts are similar within a group and differ only in magnitude; besides, the plain property lies between cool and warm (LIU et al., 2012; Liu et al., 2020). Flavors can be classified as sour, bitter, sweet, pungent, salty, plain, and astringent. Originally, the flavors referred to the taste of the medicine; however, subsequently, flavors and properties were combined to analyze the effects of the drugs (Zhang and Liu, 2015). This study showed that hypolipidemic medicinal materials were predominantly “warm and pungent” and “sweet and warm.” According to previous studies, the primary active ingredients of pungent medicinal materials are volatile oils, terpenoids, and alkaloids (Zhang and Liu, 2015), and the top three active targets with the highest correlation with pungent medicinal materials were nuclear factor erythroid 2-related factor 2 (NFE2L2), androgen receptor (AR), and prostaglandin G/H synthase 2 (PTGS2) (Chen Z. et al., 2019a). NFE2L2 is associated with atherosclerosis (Figarska et al., 2014), AR is associated with coronary artery disease (Agiannitopoulos et al., 2016), and PTGS2 is associated with myocardial infarction (Patrono, 2016). Pungent medicinal materials mainly act on the aforementioned targets and help treat cardiovascular diseases. The active ingredients of sweet medicinal materials are carbohydrate, amino acids, and vitamins (Zhang and Liu, 2015), possessing tonifying and replenishing effects (Zhang J. Y. et al., 2016). Tonifying and replenishing properties of medicinal materials can decrease blood viscosity and help in the treatment of acute cerebral infarction (Zhou D. et al., 2020). In addition, these can regulate neurotransmitters, such as serotonin and norepinephrine, to treat central nervous system diseases (Zhang X. et al., 2018). Warm and hot medicinal materials tend to promote norepinephrine release (Liu et al., 2008; Wang et al., 2014), which can increase blood flow in the coronary arteries, kidneys, brain, and myocardium (Hoekstra et al., 1990; Di Giantomasso et al., 2002) and reduce cardiovascular ischemia. According to TCM theory, pungent medicinal materials can promote blood circulation, resolve stasis, and relieving exterior syndrome by dispel heat; sweet medicinal materials can tonify qi and blood, and regulate the property of TCM in formulae. Overall, the medicinal materials with sweet and pungent flavors and warm property can promote blood circulation and help irradiate blood stasis, regulate menstruation, soothe pain (Sun et al., 2015; Zhang and Liu, 2015; Zhang J. Y. et al., 2016), and also enhance blood circulation to prevent vascular occlusion, which is consistent with the findings of modern research.

### 4.4 Analysis of Authentic and Misused Medicinal Materials

The official literature for TCM materials in Taiwan is the Taiwan Herbal Pharmacopeia, and medicinal materials recorded in the pharmacopeia and used according to TCM theory are defined as authentic drugs. During formula disassembly and literature sorting, we found that many medicinal materials collected in this study did not appear in existing pharmacopeias or used according to TCM theory and are considered to be misused medicinal materials. Further analysis of the ratio of authentic and misused medicinal materials found that the ratio of misused medicinal materials is high, showing a frequent medicinal material misuse by the Taiwanese public. Therefore, more TCM course training is required to improve the TCM identification capabilities of TCM suppliers and the public to prevent inferior medicinal materials from driving out superior medicinal materials.

### 4.5 Usage of Hypolipidemic Formulae in Taiwan

Previous studies on hypolipidemic TCM usage by Taiwanese people have collected data from the NHIRD for statistical analysis, and the hypolipidemic formulae prescribed by TCM physicians in Taiwan are mainly Xie-Fu-Zhu-Yu-Tang and Jia-Wei-Xiao-Yao-San, and hypolipidemic medicinal materials are mainly *Crataegus pinnatifida* Bunge and *Salvia miltiorrhiza* Bunge (Chu et al., 2015). As mentioned above, the trend of core hypolipidemic formulae usage found in this study was nearly identical to that of the previous study, and the difference between TCM prescription and traditional hypolipidemic formulae was that the latter additionally contained medicinal materials in Bu-Yang-Huan-Wu-Tang.

### 4.6 Study on the Hepatotoxicity of Commonly Used Medicinal Materials for Hyperlipidemia

Chinese herbal medicine is frequently used international. According to statistics from the National Center for Complementary and Integrative Health, one in five Americans used Chinese herbal medicine (National Center for Complementary and Integrative Health, 2019). However, the use of Chinese herbal medicine is mostly empirical and without safety assessment, which is the most important part in the process of western drugs development. In addition, people pay more and more attention to adverse drug reactions. Therefore, herb-induced liver injury (HILI) is gradually discussed in current researches. Referring to the previous meta-analysis study (Byeon et al., 2019), among the 17 commonly used medicinal materials in this study, there was only Cassiae semen reported to induce HILI, and the other 16 medicinal materials have not been reported to induce hepatotoxicity. In conclusion, most of commonly used medicinal materials in this study are safe.

### 4.7 Limitation

There are some limitations to this study—with regard to the selection of TCM pharmacies, although the pharmacies we visited have high local traffic and are highly representative, the distribution of these pharmacies in various counties and cities is not uniform, and most are located in the city center and easily accessible sites. Therefore, there was a slight sampling bias in this study. In future studies, the number of pharmacies sampled will be increased to evaluate hypolipidemic TCM usage more accurately in public. Considering medicinal material identification, TCM may have many origins. However, only macroscopic identification was employed in this study, and we could not determine the origins of the medicinal materials collected. In the future, a chemical identification procedure will be employed to determine the components of medicinal materials and clarify their exact origin, and the proportion of multi-origin medicinal materials used will also be discussed.

## 5 Conclusion

This study is the first ethnobotanical study that sorted and analyzed traditional hypolipidemic formulae in Taiwan. The results of this study showed that the traditional hypolipidemic formulae were similar to the fixed TCM formulae: Bu-Yang-Huan-Wu-Tang and Xie-Fu-Zhu-Yu-Tang. Besides, we recorded inherited TCM knowledge regarding hypolipidemia in Taiwan through this investigation. Although these TCMs have been used for a long time, their hypolipidemic mechanisms still remain unclear, and more studies are needed to validate their safety and efficacy.

## Data Availability

The original contributions presented in the study are included in the article/Supplementary Material, further inquiries can be directed to the corresponding author.

## Abbreviations

ACC, American College of Cardiology; AEM, *Amynthas aspergillum* (E.Perrier); AGS, *Angelica sinensis* (Oliv.) Diels; AHA, American Heart Association; AR, androgen receptor; ATG, *Astragalus mongholicus* Bunge; CAD, coronary artery disease; CKD, chronic kidney disease; CML, *Chaenomeles speciosa* (Sweet) Nakai; CMR, *Cynomorium coccineum subsp. songaricum* (Rupr.) J.Léonard; CNX, *Ligusticum striatum* DC.; CRP, *Citrus reticulata* Blanco; CSA, *Senna obtusifolia* (L.) H.S.Irwin and Barneby; CTG, *Crataegus pinnatifida* Bunge; CTL, *Cyathula officinalis* K.C.Kuan; CTM, *Carthamus tinctorius* L.; DSC, *Dipsacus inermis* Wall.; GRZ, *Glycyrrhiza uralensis* Fisch.; GTD, *Gastrodia elata* Blume; HILI, herb-induced liver injury; NCD, noncommunicable diseases; NFE2L2, nuclear factor erythroid 2-related factor 2; NHIRD, National Health Insurance Research Database; PNR, *Paeonia lactiflora* Pall.; PSC, *Prunus persica* (L.) Batsch; PTGS2, prostaglandin G/H synthase 2; RFC, relative frequency of citation; SAS, statin-associated symptoms; SVM, *Salvia miltiorrhiza* Bunge; TCM, traditional Chinese medicine; WHO, World Health Organization.
