# Supplementary material for: An Ethnopharmaceutical Study on the Hypolipidemic Formulae in Taiwan Issued by Traditional Chinese Medicine Pharmacies
Source: Front Pharmacol. 2022 Sep 15;13:900693. doi: 10.3389/fphar.2022.900693 (PMC9520573; doi:10.3389/fphar.2022.900693)
Supplement: Supplementary file 1 [file DataSheet1.docx]

**Supplementary Data**

| 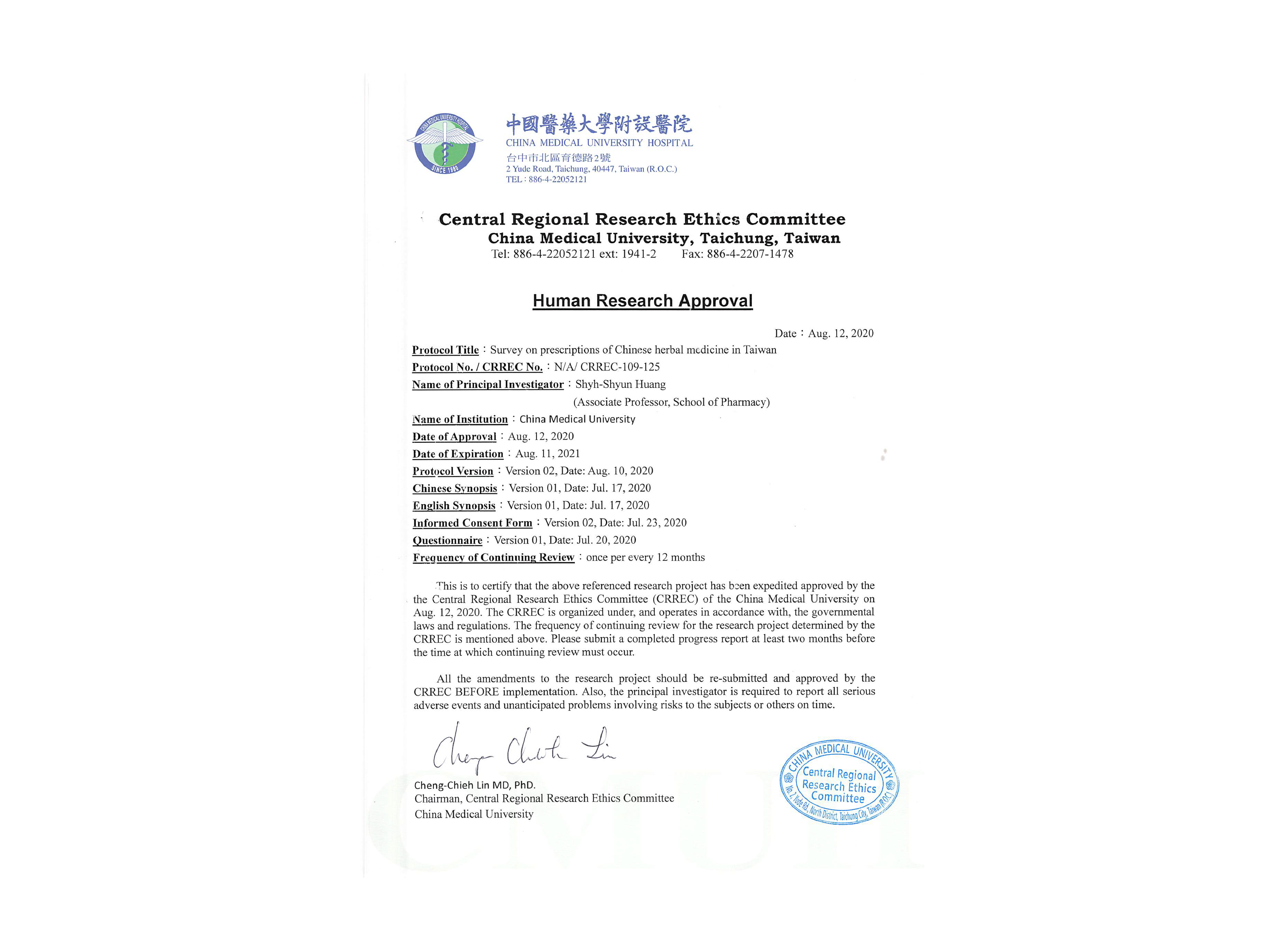 |
| --- |
| **Figure S1.** **Central Regional Research Ethics Committee of China Medical University Review Approval Letter (CRREC-109-125)** |

| 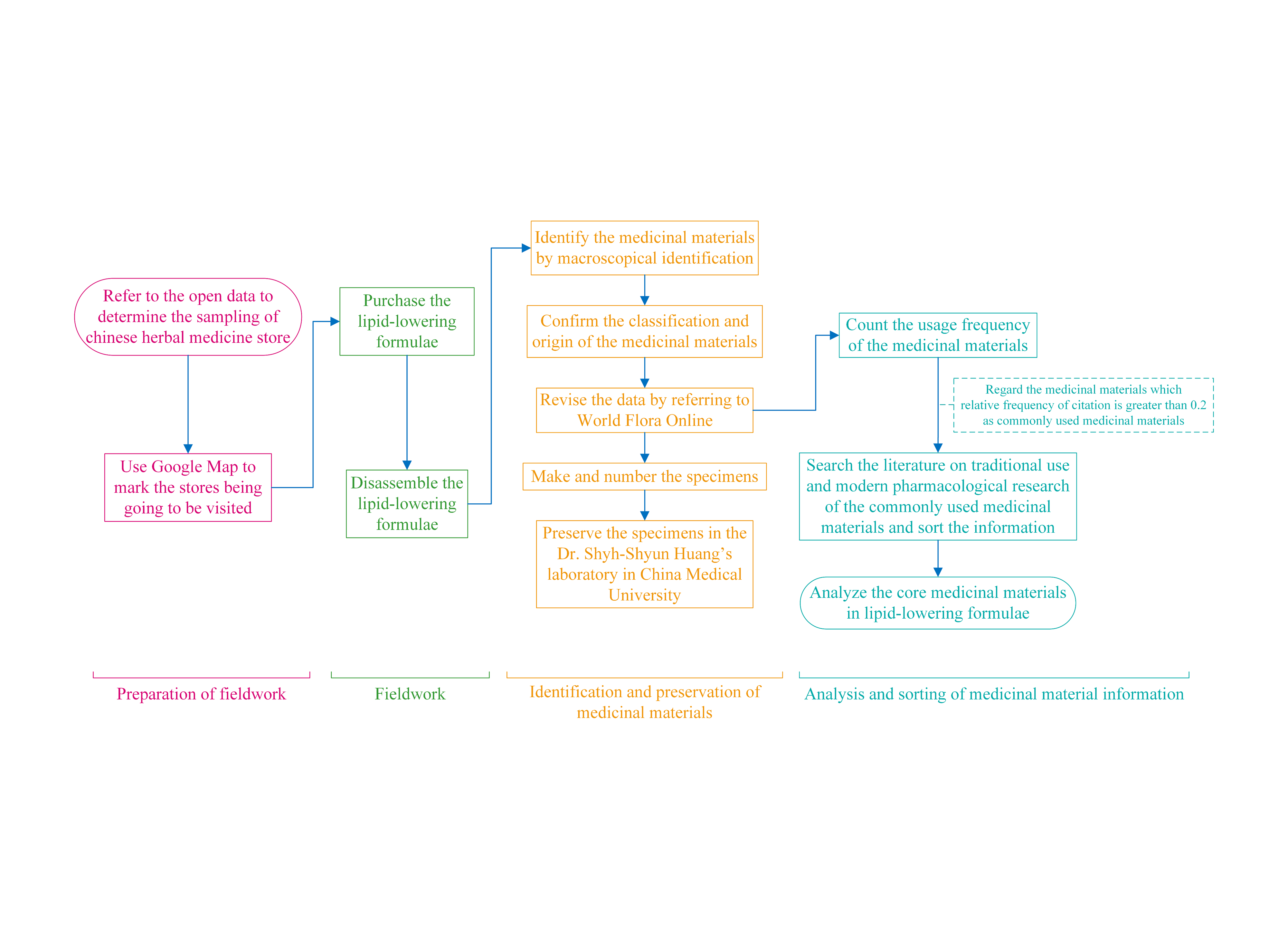 |
| --- |
| **Figure S2. Study flowchart** |
